# Supplementary material for: A Global Screen for Assembly State Changes of the Mitotic Proteome by SEC-SWATH-MS
Source: Cell Syst. 2020 Feb 26;10(2):133–155.e6. doi: 10.1016/j.cels.2020.01.001 (PMC7042714; doi:10.1016/j.cels.2020.01.001)

Q9P0W2 | HM20B_HUMAN | HMG20B BRAF35 HMGX2 HMGXB2 SMARCE1R
Monomer MW [kDa]: 35.813 Monomer expected elution fraction: 49

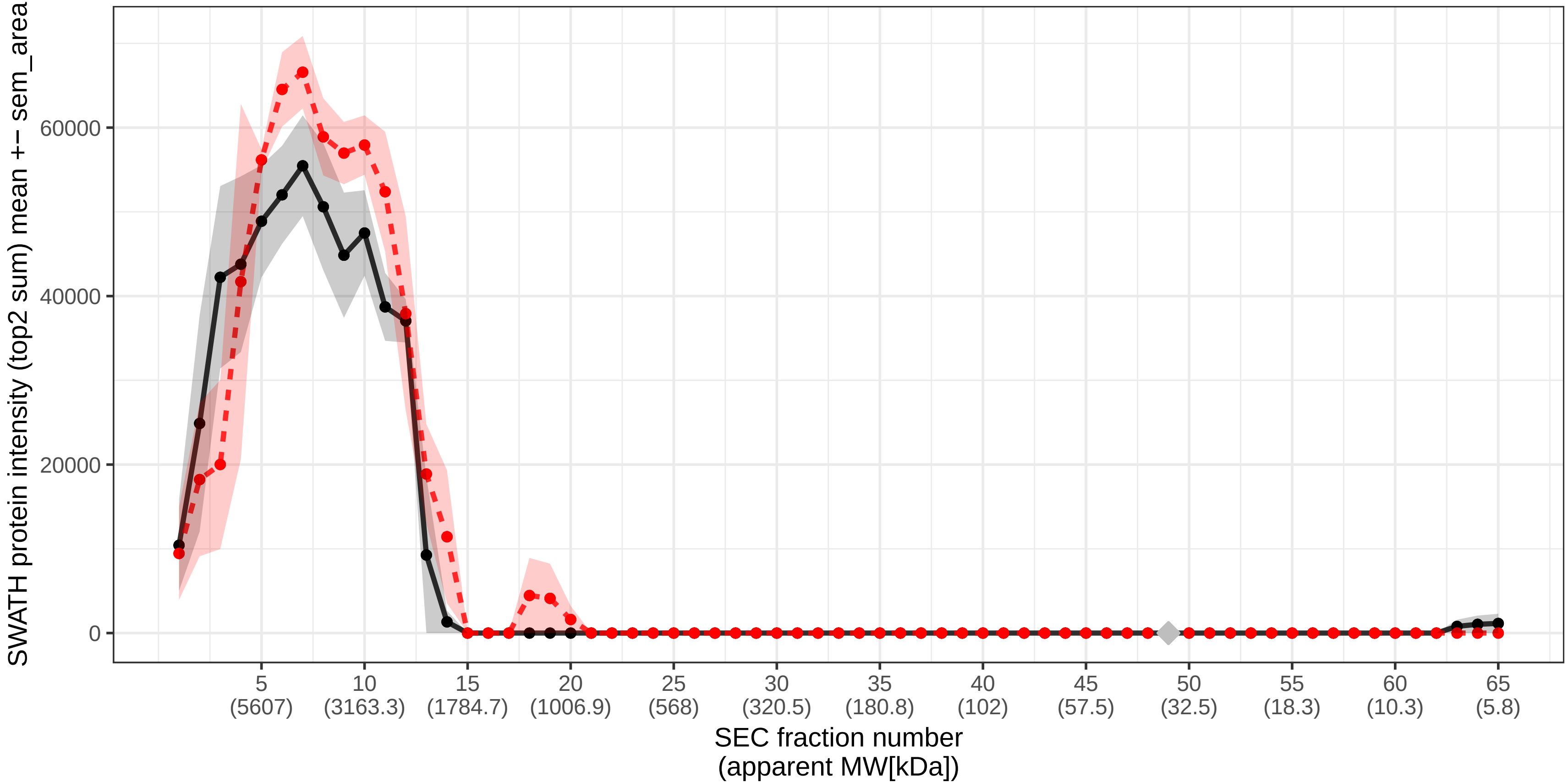

Supplement: Data S1. SEC-SWATH-MS Protein Chromatograms, Related to Figure 1 [file mmc6.zip › SECchrom_Q9P0W2_HM20B_HUMAN_HMG20B_BRAF35_HMGX2_HMGXB2_SMAR.pdf]
